# The Genetic Architecture of Intra-Species Hybrid Mito-Nuclear Epistasis

**DOI:** 10.3389/fgene.2018.00481

**Published:** 2018-11-16

**Authors:** Rania Haddad, Brandon Meter, Joseph A. Ross

**Affiliations:** ^1^Department of Biology, California State University, Fresno, Fresno, CA, United States

**Keywords:** dysfunction, speciation, incompatibility, transmission ratio distortion, linkage disequilibrium, population, X chromosome

## Abstract

Genetic variants that are neutral within, but deleterious between, populations (Dobzhansky-Muller Incompatibilities) are thought to initiate hybrid dysfunction and then to accumulate and complete the speciation process. To identify the types of genetic differences that might initiate speciation, it is useful to study inter-population (intra-species) hybrids that exhibit reduced fitness. In *Caenorhabditis briggsae*, a close relative of the nematode *C. elegans*, such minor genetic incompatibilities have been identified. One incompatibility between the mitochondrial and nuclear genomes reduces the fitness of some hybrids. To understand the nuclear genetic architecture of this epistatic interaction, we constructed two sets of recombinant inbred lines by hybridizing two genetically diverse wild populations. In such lines, selection is able to eliminate deleterious combinations of alleles derived from the two parental populations. The genotypes of surviving hybrid lines thus reveal favorable allele combinations at loci experiencing selection. Our genotype data from the resulting lines are consistent with the interpretation that the X alleles participate in epistatic interactions with autosomes and the mitochondrial genome. We evaluate this possibility given predictions that mitochondria-X epistasis should be more prevalent than between mitochondria and autosomes. Our empirical identification of inter-genomic linkage disequilibrium supports the body of literature indicating that the accumulation of mito-nuclear genetic incompatibilities might initiate the speciation process through the generation of less-fit inter-population hybrids.

## Introduction

Biological species comprise genetically related populations of organisms whose inter-population hybrid offspring suffer complete loss of fitness: either hybrid inviability or hybrid sterility (Mayr, 1942). Understanding the mechanistic process of how one formerly interbreeding population becomes two such reproductively isolated species occupies much of the contemporary study of speciation. This focus has, generally, taken the form of identifying the molecular and genetic basis of phenotypes that impede inter-population (or intra-species) mating by reducing hybrid fitness (Noor and Feder, 2006; Presgraves, 2010; Maheshwari and Barbash, 2011). Deleterious epistatic interactions between genetic variants contributed by both parents at fertilization can produce hybrid dysfunction phenotypes like hybrid inviability or hybrid sterility. These effects, by definition, reduce hybrid fitness to zero and eliminate gene flow between the nascent species. More mild hybrid dysfunction phenotypes, such as developmental delay, might also reduce gene flow.

But how can genetic differences arise and reproductively isolate two populations without first causing reduced fitness in the original populations? The DMI model addresses this concern by suggesting that neutral genetic differences independently arising in two populations could lead to deleterious epistatic interactions between two (or more) loci only when those differences were combined in inter-population hybrids (Dobzhansky, 1936; Muller, 1939, 1940). To date, DMI variants in several genes are known to cause hybrid dysfunction in a variety of taxa (Johnson, 2010; Presgraves, 2010; Maheshwari and Barbash, 2011). Although many of these DMIs involve epistasis between two nuclear alleles, others involve genetic incompatibilities between nuclear alleles and others located in the mitochondrial genome (mtDNA).

The unique relationship between the mitochondrial and nuclear genomes explains how such epistatic interactions exist and how disruption of those interactions at hybridization could be deleterious [reviewed in (Rand et al., 2004; DiMauro and Schon, 2008)]. After the genesis of mitochondria as alphaproteobacterial endosymbionts in eukaryotic cells (Sagan, 1967), many of the formerly bacterial genes, including those involved in cellular energy production, transferred to the nuclear genome (Timmis et al., 2004). The mitochondrial genome currently no longer contains all of the genes necessary for mitochondrial function, particularly with respect to oxidative phosphorylation and the production of ATP. Nuclear-encoded mitochondrial genes now contribute some of the protein subunits that comprise the five electron transport chain complexes (Blanchard and Lynch, 2000). Only one, complex II, comprises proteins encoded by genes found entirely in one genome (Hatefi and Galante, 1980; Rand et al., 2004). The other four complexes rely on functional inter-subunit interactions among genes encoded by two genomes (nuclear and mtDNA) to facilitate electron transport and ATP generation. Because of reduced effective population size (partly due to uniparental inheritance) and lack of recombination, the mitochondrial DNA is prone to mutation accumulation (Neiman and Taylor, 2009). Protein-coding changes in mtDNA thus provide an evolutionary pressure of selection for any compensatory mutations, including in genes encoding adjacent subunits (Blier et al., 2001).

As mitochondrial haplotypes (mitotypes) diverge in different populations of a species, then their nuclear genomes might fix different co-evolved alleles that sustain mitochondrial function. These co-evolved population-specific epistatic allele pairs can be disrupted at hybridization. Such a mito-nuclear epistatic interaction thus constitutes a DMI. Mito-nuclear DMI loci separated by hybridization are expected to impact electron transport and to cause hybrid dysfunction. The details of this process have been well detailed by a number of studies in various taxa where disrupting mitochondrial-nuclear (mitonuclear) epistatic interactions by hybridization causes fitness reduction (Breeuwer and Werren, 1995; Sackton et al., 2003; Fishman and Willis, 2006; Bolnick et al., 2008; Ellison et al., 2008; Lee et al., 2008; Burton et al., 2013; Meiklejohn et al., 2013; Spirek et al., 2014; Barnard-Kubow et al., 2016; Chang et al., 2016; Ma et al., 2016; Du et al., 2017; Lamelza and Ailion, 2017; Barreto et al., 2018). This growing body of literature demonstrates a recurring role of mito-nuclear DMIs that potentially initiate speciation.

Mitochondrial-nuclear DMIs might also help explain Darwin’s corollary to Haldane’s rule (Turelli and Moyle, 2007): the asymmetry observed in the reduced viability or fecundity of one sex in hybrids. Asymmetric fitness effects in reciprocal crosses require deleterious epistatic interactions (such as DMIs), because only uniparentally inherited factors (e.g., cytoplasmic genomes, sex chromosomes, epigenetic marks, maternal or paternal effects) will differentially affect F1 hybrids (Turelli and Orr, 2000; Turelli and Moyle, 2007). Asymmetric DMIs involving uniparentally inherited genetic factors exist in diverse taxa, e.g., (Breeuwer and Werren, 1995; Bolnick et al., 2008; Lee et al., 2008; Chang et al., 2016). Thus, genetic factors that are more likely to negatively impact hybrid fitness, and thus to participate in reproductive isolation, include sex chromosomes and cytoplasmic genomes such as mtDNA (Rand et al., 2001).

Loci involved in hybrid dysfunction can be identified by genotyping hybrids to identify allele combinations that are selected against due to the reduced fitness they produce. These combinations are then underrepresented in hybrids compared to the Mendelian expectation. Genotypes of hybrids can be assessed for TRD at loci across the genome to map incompatibility loci (Moyle and Graham, 2006; Kao et al., 2010; Nadeau, 2017). To accommodate the identification of potentially complex genetic incompatibilities, it can be valuable to use recombinant inbred lines (RIL) to detect DMI loci because of the finer haplotype block structure that RIL contain (Colome-Tatche and Johannes, 2016; Ehrenreich, 2017).

In order to assess the contribution of cytoplasmic alleles to hybrid dysfunction, genetic mapping often also employs reciprocal crosses, with the intent of identifying phenotypes that depend on interactions between maternally inherited cytoplasmic factors (such as the mitochondrial genome) and a hybrid nuclear genome. Cytoplasmic-nuclear hybrids (cybrids), in which the cytoplasm from one population is introgressed into the nuclear background of another, have revealed mito-nuclear epistatic interactions that decrease cybrid fitness in several taxa (Sackton et al., 2003; Fishman and Willis, 2006; Arnqvist et al., 2010; Meiklejohn et al., 2013; Spirek et al., 2014; Ma et al., 2016; Lamelza and Ailion, 2017). Such findings have suggested that mitochondrial and nuclear genetic variation plays an important role in generating hybrid dysfunction that would initiate or reinforce hybrid genetic incompatibilities and therefore speciation (Gershoni et al., 2009; Johnson, 2010; Burton and Barreto, 2012; Hill, 2015).

Intra-species cybrids of the nematode *Caenorhabditis briggsae* have also revealed mitonuclear epistasis. Like its close relative, *C. elegans*, it could be considered a non-traditional model for studying speciation genetics, because the presence of a hermaphrodite sex and only rare males means that each wild population is produced predominantly by selfing. Thus, these populations are essentially functionally reproductively isolated from others. However, *C. briggsae* populations are generally reproductively compatible with other populations when mated in the laboratory (Dolgin et al., 2008), thus adhering to the classical definition of the biological species concept. We recently confirmed the presence of deleterious mitochondria-nuclear (mito-nuclear) epistatic interactions in hybrids between two wild populations of *C. briggsae*, named AF16 and HK104. Experimental AF16-HK104 cybrid lines exhibit decreased fecundity, increased reactive oxygen species levels, and an increase in lipid content compared to the wild isolate parental populations (Chang et al., 2016).

The existence of AF16-HK104 mito-nuclear epistasis led us to further explore the genetic architecture of this interaction, with the long-term goal being the identification of the incompatible alleles. Our efforts involved creating and genotyping AF16-HK104 hybrid lines to observe TRD and thus to identify epistatic loci potentially under selection in *C. briggsae* hybrid lines. After identifying combinations of alleles underrepresented in the lines, we also assessed the presence of asymmetric TRD. We found that loci whose inheritance patterns were most skewed from the neutral expectation were located on chromosome III and the X chromosome. Asymmetries were also observed, most notably on the X. Our data suggest that genetic variants involving mitochondrial DNA and the X chromosome in wild populations of *C. briggsae* might comprise a mito-nuclear DMI. These loci could contain the types of genetic variants that initiate the process of speciation by decreasing hybrid fitness.

## Materials and Methods

### Strain Husbandry

Samples of the wild isolate *C. briggsae* populations AF16 and HK104 were obtained from the Caenorhabditis Genetics Center. Nematodes were cultured on NGM plates with *Escherichia coli* strain OP50 as a food source according to (Stiernagle, 2006). The populations were maintained in a digitally controlled incubator at 20.0°C.

### Advanced-Intercross Recombinant Inbred Lines (AI-RIL)

**FIGURE 1:**
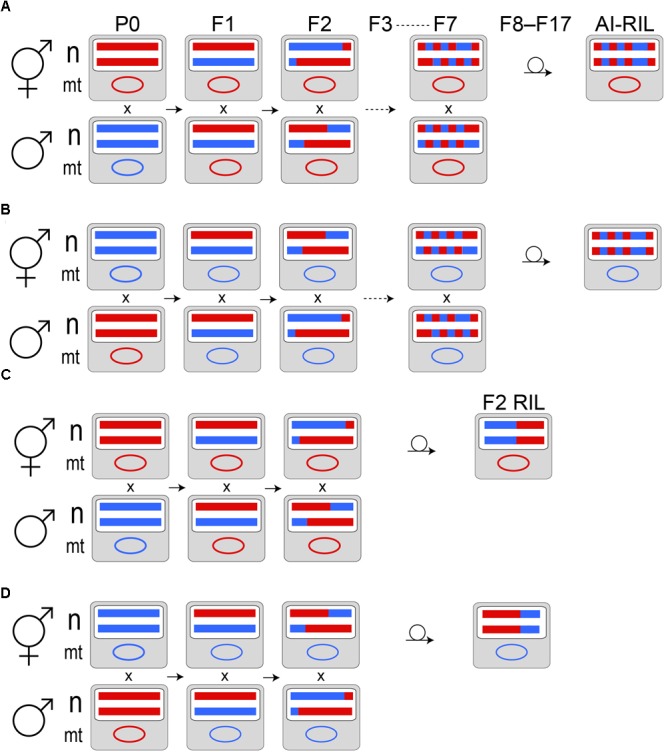
Cross designs. In the P0 generation, a self-sperm depleted hermaphrodite (pseudofemale) of one *C. briggsae* population is mated to males from another population. Example nuclear (n) homologs and a mitochondrial (mt) genome are depicted as colored horizontal lines and an oval, respectively. Haplotypes from AF16 are red; those from HK104 are blue. Panels **(A,B)** represent the AI-RIL cross design in reciprocal directions: **(A)** depicts the HK104 × AF16 cross, while **(B)** depicts the AF16 × HK104 cross (by convention, the male population is written first, followed by the pseudofemale population). Maternal mitochondrial inheritance is expected. Because the P0 individuals are generated from populations that propagate by selfing, their nuclear genomes are completely homozygous. F1 hermaphrodite offspring, which are thus completely heterozygous in the nuclear genome, are mated to male siblings to produce the F2 generation. Because of extensive crossover interference in Caenorhabditis (Hillier et al., 2007), we expect, on average, that meiotic recombination introduces a single haplotype breakpoint in each of the F1 through F7 generations. Using a single F7 hermaphrodite, each replicate line is passaged by selfing a single hermaphrodite per generation for ten generations to produce an AI-RIL. Each AI-RIL then has a hybrid nuclear genotype that is expected to be homozygous at each locus. Panels **(C,D)** represent the F2 RIL cross design in reciprocal directions: **(C)** depicts the HK104 × AF16 cross, while **(D)** depicts the AF16 × HK104 cross.

Hybrid lines were produced by P0 generation crosses AF16xHK104 and HK104xAF16 (male listed first, hermaphrodite listed second). Because of the presence of a self-fertile hermaphrodite sex in *C. briggsae*, hermaphrodites used in crosses were all “pseudo-females:” worms that were purposefully raised in order to eliminate their self-sperm and thus to ensure that only cross-progeny were produced after the introduction of males from a different population. Each hybrid line was established in the F1 generation, for each biological replicate of each of the two crosses above, from three sibling-mated (as determined by presence of a copulatory plug) hermaphrodites, as in (Ross et al., 2011). Seven such generations of sibling-mating (F1–F7) were followed by ten generations of inbreeding by single hermaphrodite selfing to render each line homozygous throughout the nuclear genome (Figure 1; Ross et al., 2011). After genomic DNA extraction was performed, all of the AI-RIL were lost to desiccation prior to cryopreservation using the liquid freezing protocol (Stiernagle, 2006). Purified genomic DNA samples from these lines do still exist.

### F2 Recombinant Inbred Lines (F2 RIL)

Like the AI-RIL, lines were initiated by P0 generation crosses AF16xHK104 and HK104xAF16. Each hybrid line was established in the F1 generation, for each biological replicate of each of the two crosses above, by one sibling-mated hermaphrodite. One additional generation of sibling-mating occurred, followed by ten generations of inbreeding by single hermaphrodite selfing. All of the F2 RIL were cryopreserved using the liquid freezing protocol (Stiernagle, 2006).

### Polymerase Chain Reaction (PCR) Genotyping

Phenol-chloroform extracted and isopropanol-precipitated genomic DNA samples were resuspended and then diluted to 5 ng/μL concentration in 10mM TRIS buffer pH 8.0. Amplification was performed according to (Chang et al., 2016) with the following modifications. Amplified fragment length polymorphism (AFLP)- and restriction fragment length polymorphism (RFLP)-amplifying primer sequences were obtained from (Koboldt et al., 2010) and synthesized by Integrated DNA Technologies (Coralville, IA, United States). The AFLP genotyping assays are named: cb-m26, cb-m56, cb-m205, cb-m161, cb-m172, cb-m103, cb-m197, cb-m124, and cb-m127. The RFLP assays are cb18178 and cb51757. The relevant restriction enzymes were obtained from New England Biolabs (Ipswich, MA, United States); digest of PCR amplicons was conducted according to the manufacturer’s suggested protocols.

### Statistical Analysis

Observed allele TRD in the AI-RIL was compared to the neutral expectation using chi-square tests with alpha = 0.05. For each cross direction, TRD was calculated as the ratio of the number of HK104 alleles to the total number of alleles (for a diploid organism like *C. briggsae*, this value is equal to twice the number of replicate lines) (Ross et al., 2011). The null hypothesis for autosomal allele retention of hybrid lines in the absence of selection is that half of replicate line alleles will represent one P0 population allele and half the alleles from the other P0 population. Because of X-chromosome dosage differences between the sexes, the expected allele retention depends on cross direction: one-third for the paternal X allele and two-thirds for the maternal X allele. Analyses were Bonferroni-corrected for multiple tests (ten markers x two cross directions = twenty tests) to a significance level of *p* < 0.0025.

## Results

Thirty-nine hybrid lines initiated by reciprocal crosses between AF16 and HK104 (nineteen AF16xHK104 and twenty HK104xAF16) completed the AI-RIL cross design (Figure 1 and Table 1 “Experiment 1”). Twenty-one hybrid lines (nine AF16xHK104 and twelve HK104xAF16) completed the F2 RIL cross design (Table 1 “Experiment 2”).

**Table 1:** RIL transmission ratio distortion (TRD) values. For each of two experiments, between nine and twenty biological replicate RIL were produced in reciprocal cross directions.

Experiment	Hybrid	Male	Hermaphrodite	Replicates	HK104 allele fraction on X
1	AI-RIL	AF16	HK104	19	1.00 (cb-m127)^∗^
	AI-RIL	HK104	AF16	20	0.00 (cb-m127)^∗^
2	F2 RIL	AF16	HK104	9	0.44 (cb51757)
	F2 RIL	HK104	AF16	12	0.08 (cb51757)^∗^

*The two experiments differ (i) by the use of AI-RIL in experiment 1 and F2 RIL in experiment 2 and (ii) in the number of replicates produced. TRD values correspond to the fraction of HK104 alleles in replicate RIL at one locus on the X (either cb-m127 or cb51757). TRD values that are statistically significantly different from the null hypothesis (0.66 for AF16xHK104 crosses; 0.33 for HK104xAF16 crosses) are indicated (^∗^Bonferroni-corrected chi-square test).*

### AI-RIL Genotypes

**FIGURE 2:**
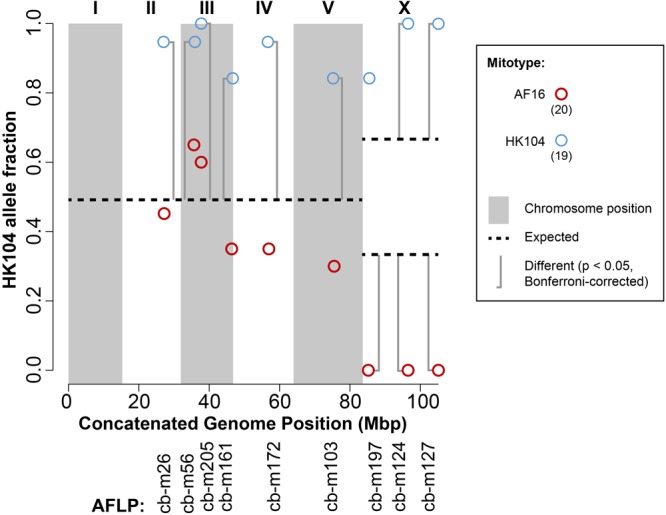
Transmission ratio distortion (TRD) in AF16-HK104 AI-RIL. AFLP genetic markers are listed on the x-axis according to their chromosomal coordinates after concatenating all chromosome sequences into a single sequence. Alternating gray and white vertical shading indicates the extents of chromosomes I-V and the X chromosome. The fraction of HK104 alleles present in all replicates of each of the two crosses (AF16xHK104 and HK104xAF16) is plotted on the y-axis. Blue circles represent data from lines with HK104 mitotypes, and red circles represent data from lines with AF16 mitotypes. The number of replicates of each cross type is given in parentheses in the figure key. Horizontal dashed lines indicate the null expectation for HK104 allele TRD in the absence of selection or drift (0.5 for autosomes and either 0.66 or 0.33 for the X chromosome, depending on cross direction). Vertical brackets indicate comparisons between observed and expected values that are statistically significantly different (Bonferroni-corrected chi-square test).

Purified genomic DNA from each AI-RIL was PCR genotyped at nine indel markers distributed across four of the five autosomes and the X chromosome, and at one RFLP marker in the mitochondrial genome (cb18178). The mitotypes were all maternally inherited, as expected, with no evidence of heteroplasmy. The fraction of HK104 alleles present at each nuclear marker is shown in Figure 2. For the nineteen replicates containing the HK104 mitotype, eight of the nine nuclear markers show significant bias toward retention of HK104 alleles. Two of the three markers on the X chromosome show absolute bias (in that AF16 alleles were completely excluded). For the twenty replicates with the AF16 mitotype, the three markers on the X chromosome reveal a significant and absolute bias toward retention of AF16 alleles.

### F2 RIL Genotypes

To validate AI-RIL genotype results, purified genomic DNA from each F2 RIL was PCR genotyped at a different marker on the X chromosome: cb51757. The fraction of HK104 alleles present at this X locus is shown in Table 1. For the nine replicates containing the HK104 mitotype, no significant TRD was observed. For the twelve replicates containing the AF16 mitotype, a significant bias exists for the retention of AF16 alleles on the X chromosome.

## Discussion

We explored the genetic architecture of mito-nuclear epistasis in *C. briggsae* hybrids. Despite determining genotypes at a limited number of loci across most (but not all) nuclear chromosomes, our results are consistent with a number of themes. First, cross direction, and thus possibly mitotype, modulates some TRD patterns. Second, a nuclear chromosome consistently implicated in inter-genomic epistasis was the X chromosome. Below we interpret this result according to theory that suggests the possibility of an increased role of an X chromosome in mito-nuclear interactions. Third, mito-nuclear TRD appears to reveal co-evolution. We discuss these findings in relation to the view that mito-nuclear DMIs play an important role in the process of speciation (Johnson, 2010; Burton and Barreto, 2012).

### TRD Patterns in AI-RIL

The nineteen AI-RIL with HK104 mitotypes always retained HK104 X chromosome alleles at cb-m124 and cb-m127 (Figure 2); such a pattern supports the interpretation that mito-X co-evolution has occurred in HK104. We also observed equally strong mito-X epistasis in HK104xAF16 AI-RIL, where all twenty replicate lines were devoid of HK104 alleles at all three X chromosome markers we genotyped (Figure 2). We interpret this pattern as evidence for co-evolution between the AF16 mitotype and AF16 X chromosome alleles.

With regard to the autosomal TRD data, in the nineteen AI-RIL with HK104 mitotypes, we also observed a significant bias toward retention of HK104 alleles at every autosomal marker we tested (Figure 2). Although our panel of markers provides minimal coverage, this potentially genome-wide TRD further gives the appearance of co-evolution between mitotype and nuclear genotype in HK104.

One significant pattern of TRD in the AF16-HK104 AI-RIL involves chromosome III. The genotypes at cb-m205 reveal bias toward retention of HK104 alleles in all crosses except those with AF16 mitotypes at 20°C. We suspect that this TRD, which is independent of temperature and of mitotype, is related to a complex dysgenic interaction in AF16-HK104 hybrids that causes F2 larval developmental delay in individuals homozygous for AF16 alleles on the middle of chromosome III (Ross et al., 2011; Baird and Stonesifer, 2012). Thus, a bias toward retention of HK104 alleles on chromosome III was expected. Hybrid developmental delay is one mechanism by which Dobzhansky-Muller incompatibilities (DMIs) might reduce hybrid fitness, by decreasing the ability of individuals with particular genotypes to compete against their wild-type siblings for resources (e.g., food) that facilitate propagation of those genotypes. Other phenotypic effects of DMIs could explain TRD. For example, we did not measure the fecundity or embryonic lethality of our hybrid lines across their production, but it is possible that these could be selective forces driving the TRD we observe, as AF16-HK104 hybrids are known to exhibit increases in both of these phenotypes (Dolgin et al., 2008).

### TRD Patterns in F2 RIL

In order to replicate our observations of epistasis in AF16-HK104 AI-RIL, we created additional hybrid lines using an F2 RIL cross design. Although this design is less powerful for mapping and more prone to the effects of drift, we chose to use this design because such lines are more efficient to produce. Because of the extreme TRD we observed on the X chromosome in AI-RIL, and to reduce multiple-testing effects, we opted to focus our analysis on measuring TRD only at one X chromosome in the F2 RIL. We also selected a new locus on the X chromosome at which to confirm TRD: cb51757, which is located at 12.0 Mbp on the X chromosome sequence assembly. In the F2 RIL containing AF16 mitotypes, a TRD pattern similar to that from the AI-RIL was observed: a significant bias toward retention of AF16 alleles on the X chromosome. No significant bias was observed in the reciprocal cross.

### Comparing F2 and AI-RIL TRD

On the X chromosome, the AI-RIL showed absolute TRD at two of three loci measured (Figure 2: cb-m124 and cb-m127). In the F2 RIL, no absolute TRD was observed. In one cross direction (HK104 x AF16), the AI-RIL and F2 RIL TRD was biased in the same direction, each with a significant difference from the expected value (Table 1). In the reciprocal cross, however, despite finding significant TRD in the AI-RIL, we observed no statistically significant difference from expected in the F2 RIL. Here we present possible explanations for how i) the data from F2 RIL do not always exhibit the same TRD as the AI-RIL, and ii) the F2 RIL TRD values are not as strong as those from the AI-RIL.

First, it is possible that, given our cross designs and number of biological replicates, drift might have influenced our TRD values (discussed in greater detail below). We favor the interpretation that differences between the AI-RIL and F2 RIL cross designs account for some observed discrepancies between our datasets. In the AI-RIL cross, recombinant genotypes have opportunity to compete in small populations (reducing the influence of drift), while this did not occur in the F2 RIL cross, where drift might dominate over all but the most severe fitness effects. We previously showed, using different AF16-HK104 F2 RIL and AI-RIL, that this is the case (Ross et al., 2011). In that previous study, F2 RIL only revealed TRD on chromosome III, owing to the strong effect of selection against F2 hybrids with AF16 homozygosity; AI-RIL revealed the same TRD on chromosome III but also allele retention bias on other chromosomes as well. Measurements of TRD in AI-RIL more finely reflect incompatibilities because of their smaller haplotype blocks, which are likely to enable the creation of more maladapted allele combinations than in F2 RIL (Ross et al., 2011). Thus, even F2 RIL can detect selection acting above any effects of drift using such a cross design. We suggest that, because F2 RIL are less sensitive to hybrid incompatibilities, it is not surprising that the AF16xHK104 RIL did not produce a significant bias, as did the corresponding AI-RIL.

Second, we note that the F2 RIL had the fewest biological replicates, which could also influence the ability to detect a significant and consistent signal of TRD. In fact, we find it remarkable, then, that we did observe significant X-chromosome TRD in the HK104xAF16 RIL. This result from F2 RIL, which corroborates the TRD bias in AI-RIL with the same mitotype, raises the possibility that mito-X co-evolution constitutes a strong selective pressure to maintain linkage disequilibrium (LD) between alleles from the same population.

### Comparisons With Prior Findings

Most of our observations corroborate previous TRD data from other *C. briggsae* AF16-HK104 hybrid lines. Genotypes of AF16-HK104 F2 RIL have shown a slight tendency for inheritance of HK104 alleles over AF16 across the autosomes and X chromosome (Hillier et al., 2007). Further, in AF16-HK104 AI-RIL, similar biases were evident (Ross et al., 2011). In particular, loci in the middle of chromosomes III, IV and V were significantly biased toward HK104 allele retention. However, in neither of these studies were any pair of loci on different chromosomes found to be in complete LD. This suggests that, unlike the case of *zeel-1* and *peel-1* in *C. elegans* (Seidel et al., 2008; Rockman and Kruglyak, 2009; Seidel et al., 2011), there are no two-locus lethal allele combinations in AF16-HK104 hybrids.

Our present data from AF16-HK104 hybrid lines exhibit similar patterns to those reported previously, with most autosomes and the X biased toward retention of HK104 alleles. However, the strength of the TRD does differ. In our AI-RIL with HK104 mitotypes, we observed, in nineteen replicate lines, complete LD between HK104 alleles in the middle of chromosome III and HK104 alleles on the X chromosome. This effect has a greater magnitude than in other AF16-HK104 AI-RIL (Ross et al., 2011). Further, in our F2 RIL, we observed a stronger bias on the X (8% HK104 allele fraction) than previously reported (just over 50% HK104 allele fraction) (Baird et al., 2005; Hillier et al., 2007). We intentionally created our AI-RIL and F2 RIL, as best we could, with the cross designs reported in those prior studies. Selection against a known developmental delay phenotype in AF16-HK104 F2 hybrids likely explains the observed TRD on chromosome III (Ross et al., 2011; Baird and Stonesifer, 2012). Thus, we must address why our present TRD observations on the X chromosome might be as extreme as they are. The most obvious differences between our AI-RIL and those used previously are that we generated many fewer replicate lines and also genotyped many fewer loci. Because of our smaller dataset, we are concerned primarily with the possibility that drift or another random effect might have contributed to the stronger TRD signal we report here. However, we argue that such an effect is not likely the cause of our results.

### A Possible Role of Drift in TRD

Although our RIL cross design involved small populations sizes, which enhance the action of drift in allele fixation, drift is not likely to account for the TRD patterns we observed. If drift, and not selection, were the dominant force in driving TRD, then we would expect an unbiased pattern of allele fixation: on average, half of replicate lines for any particular cross would fix one allele and half of the lines would fix the other allele. This is because all replicate lines only share the same genotype at the F1 generation, when all loci in the nuclear genome are heterozygous. So, every line had an equal probability of fixing the AF16 or the HK104 allele in the absence of selection. As such, for the marker where we have the most power (X chromosome, cb-m124, where 20 AF16xHK104 replicate lines all fixed the HK104 allele and 20 HK104xAF16 replicate lines all fixed the AF16 allele), the probability of drift resulting in these observations from 20 lines is, conservatively, 0.5ˆ20 = 9.5 × 10-7.

Interestingly, alleles on chromosomes III and X (cb-m205 and cb-m124, for example) are in perfect LD with the HK104 mitotype and also share a TRD pattern with loci on chromosomes II, IV and V. Using the same probability argument, it would be very unlikely for drift to have caused significant TRD on five chromosomes toward retention of the same allele (HK104), in the case of the AI-RIL with HK104 mitotypes (Figure 2). Even if each of the allele fraction values were from the genotype of only a single line, then the probability of a random force like drift biasing toward retention of the HK104 allele on five nuclear chromosomes is approximately 0.5ˆ5 or 3.1%. Although this is a relatively small probability, it is also a conservative one, because each allele fraction value is based on observations from multiple replicate lines and because on some chromosomes (III, X) we have additional markers that also show consistent TRD bias toward HK104 allele retention. Such a pattern suggests the existence of nuclear genome-wide epistasis.

We also note it is possible that positive epistasis between the HK104 X allele and the cb-m205 allele, and not with the HK104 mitotype, explains the large HK104 allele fraction values on chromosomes III and X. Even if true, then strong evidence for mito-X epistasis still exists in the AI-RIL with AF16 mitotypes, where we observed no other nuclear alleles in LD with the X, although our sparse nuclear genotyping might not have identified other nuclear loci whose AF16 alleles could theoretically interact positively with the X allele to result in the extent of X TRD we observed. At present, the correlation of X allele TRD with mitotype is not likely influenced by drift and also is consistent with the existence of mito-X epistasis.

On balance, the F2 cross design we used was not optimal for high-resolution mapping and detection of weak interactions. However, in the F1 hybrid generation for both cross types (F2 RIL and AI-RIL), each mitotype exists in the same (heterozygous) nuclear background. Any subsequent observed differences between reciprocal crosses thus correlate with that singular genetic difference between reciprocal crosses: their mitotypes. The detection of a consistent signal of TRD on the X chromosome in F2 and AI-RIL, and the strong TRD signals observed using multiple biological replicate lines, including across three loci spanning two chromosomes, do support the possibility that a mito-X epistatic interaction exists and possibly signals the co-evolution of alleles within each population.

### Epistasis and the X Chromosome

Alleles that experience positive epistatic interactions should be transmitted together and thus, because of mito-nuclear co-evolution (Rand et al., 2004), mitochondrial haplotypes would optimally be inherited with interacting nuclear-encoded mitochondrial alleles (Hill, 2015). Thus, the X chromosome provides an ideal nuclear locus for genes to evolve alleles that are co-adapted with cytoplasmic factors (Rand et al., 2004; Rogell et al., 2014). The population genetics of X chromosomes and mitochondria suggest why such a trend might be observed. As mitochondria tend to be maternally inherited, and as X chromosomes are more frequently present in females (particularly in androdioecious Caenorhabditis species, where males are rare in nature) (Ward and Carrel, 1979; Hodgkin and Doniach, 1997), co-evolution between the mitochondrial and nuclear genomes might preferentially occur between mitochondria and X chromosomes to result in female-beneficial epistatic interactions (Rand et al., 2001; Gemmell et al., 2004; Patel et al., 2016). In our intra-species *C. briggsae* AI-RIL, we observed that mitochondria preferentially segregate with X alleles from the same population. This finding indicates that a separate effect, deleterious epistasis between AF16 and HK104 X alleles, is unlikely to explain the X chromosome TRD (Matute and Gavin-Smyth, 2014). It is possible that these effects of hybridization on TRD result from selection acting during the generation of our hybrid lines to maintain co-evolved mito-X alleles, although as noted above it is also possible that X-autosome or autosome-mitochondria epistasis between alleles from the same population could contribute to this effect. With regard to interpretation of autosomal TRD, two additional *caveats* should be noted. We only genotyped one locus per autosome and did not include chromosome I. Also, Caenorhabditis species have only five autosomes. Thus, we are not attempting to conclude here whether mitochondrial epistasis with X alleles is more pervasive or deleterious in our RIL than epistasis involving autosomal loci.

### The Symmetry of Epistatic Interactions

Theoretical work, supported by organismal data, has suggested that asymmetrical fitness effects (with respect to cross direction) result from genetic incompatibilities between nuclear and parent-of-origin (e.g., cytoplasmic genome, maternal effect) sources and that symmetrical effects could result from presence of multiple DMIs or from the separation of co-evolved mito-nuclear loci (Turelli and Orr, 2000; Turelli and Moyle, 2007). Although we cannot rule out the possibility that symmetric epistatic effects in *C. briggsae* AI-RIL might result from multiple DMIs, the observation of asymmetric effects (Figure 2) further implicates a cytoplasmic source, possibly the mitochondrial genome, as a partner in an inter-genomic epistatic interaction. The observation of epistasis involving the X chromosome in our replicate experiments possibly results from the maladaptive recombination of co-evolved mito-X loci representing a DMI pair. Recent empirical studies have shown that many DMIs are asymmetric, e.g., (Brideau et al., 2006; Ferree and Barbash, 2009), and that mito-nuclear DMI can either be asymmetric (Lee et al., 2008; Chang et al., 2016) or symmetric (Lamelza and Ailion, 2017). Here, mito-X TRD in *C. briggsae* AI-RIL is symmetric (i.e., occurs in both cross directions), while the autosomal retention of HK104 alleles in the presence of the HK104 mitotype is asymmetric. In this context, it is interesting to note that crosses involving species containing a hermaphrodite sex (i.e., “selfers”), as is true in the present study, have been suggested to generate larger effect asymmetries than in dioecious (male-female) species (Turelli and Moyle, 2007).

## Conclusion

The study of mito-nuclear epistatic interactions segregating within populations offers the opportunity to better understand the genetic basis of incipient speciation (Chou and Leu, 2010; Johnson, 2010; Burton and Barreto, 2012; Hill, 2015). With both intra-specific and inter-specific genetic incompatibilities segregating in *C. briggsae* populations, this species is ideal for studying speciation genetics (Woodruff et al., 2010; Kozlowska et al., 2012; Yan et al., 2012; Bi et al., 2015), especially with respect to the involvement of mitochondria in generating deleterious epistatic interactions in hybrids. Studies in mosquitoes and Drosophila have suggested an important role of mito-X epistasis in creating asymmetrical DMIs (Coyne and Orr, 1989; Presgraves and Orr, 1998). Others have previously identified, in genera like Caenorhabditis, Drosophila, Mimulus, Mus, Saccharomyces and Solanum (Presgraves, 2003; Kao et al., 2010; Turner and Harr, 2014; Garner et al., 2016; Guerrero et al., 2017), that although many incompatibility loci are scattered throughout the nuclear genome, such loci are sometimes more concentrated on the X chromosome than autosomes (True et al., 1996; Bi et al., 2015). Further, X chromosome variants have been implicated in speciation in taxa including fruit flies and crickets (Cabot et al., 1994; Maroja et al., 2015). Although these studies support our preferred interpretation of TRD data, the likelihood of the X chromosome playing an integral role in speciation in the Caenorhabditis genus has been unclear (Cutter, 2017). Synteny across the genus is greater on the X than autosomes, particularly owing to fewer inversions (Ross et al., 2011; Fierst et al., 2015). However, the X might carry a higher mutational load (Thomas et al., 2015); those mutations presumably could generate more DMIs. Additional studies suggested that the X plays a disproportionately large role in hybrid incompatibility between Caenorhabditis species like *C. remanei* and *C. latens* (Bundus et al., 2018) and *C. briggsae* and *C. nigoni* (Bi et al., 2015). The present study adds to this body of literature by describing the basic genetic architecture of mito-nuclear epistasis, possible involving a mito-X DMI, in *C. briggsae*.

In future, we will identify the epistatic loci and describe the organismal fitness effects that result in the allele TRD reported here. Improving our understanding of the mechanics of epistasis is important (Ehrenreich, 2017). Initial work to identify the DNA sequence differences underlying epistatic interactions focused on allele pairs, e.g., (Brem et al., 2005), but more complex interactions exist (Taylor and Ehrenreich, 2015). Although the relative contribution of epistasis to the heritability of quantitative traits is debated (Huang and Mackay, 2016), understanding how alleles interact with each other will, for example, improve our ability to predict phenotype from genotype (Forsberg et al., 2017), which is one of the National Science Foundation’s Five Grand Challenges in Biology: “Understanding the Rules of Life: Predicting Phenotype.” Further, understanding effects of genetic background on novel genetic variants, e.g., (Paaby and Rockman, 2014), could be as important for mitochondrial haplotypes as for nuclear variants given the advent of three-parent fertilization and the possibility of deleterious mito-nuclear epistasis (Kang et al., 2016; Eyre-Walker, 2017), such as that demonstrated in intra-species mitochondrial replacement embryonic lethality in mice (Ma et al., 2016). A recent study also demonstrated the extent to which sexually antagonistic mito-nuclear interactions exist in Drosophila populations (Camus and Dowling, 2018). Such studies, including ours in *C. briggsae*, continue to motivate the exploration of how mito-nuclear epistasis influences fitness and, ultimately, speciation.

## Data Availability Statement

The raw agarose gel electrophoresis images supplying the genotype data and the nematode strains or purified genomic DNA samples supporting the conclusions of this manuscript, will be made available by the authors, without undue reservation, to any qualified researcher.

## Author Contributions

RH created the AI-RIL and BM created the F2 RIL. RH and BM isolated and genotyped DNA samples from the RIL, analyzed the data, and conducted statistical analyses. RH and JR wrote the manuscript.

## Conflict of Interest Statement

The authors declare that the research was conducted in the absence of any commercial or financial relationships that could be construed as a potential conflict of interest.

## Abbreviations

AI-RIL: advanced-intercross recombinant inbred line
DMI: Dobzhansky-Muller Incompatibility
TRD: transmission ratio distortion
